# Signals That Trigger Dendrite Growth Are Identified

**DOI:** 10.1371/journal.pbio.1001154

**Published:** 2011-09-20

**Authors:** Richard Robinson

**Affiliations:** Freelance Science Writer, Sherborn, Massachusetts, United States of America

**Figure pbio-1001154-g001:**
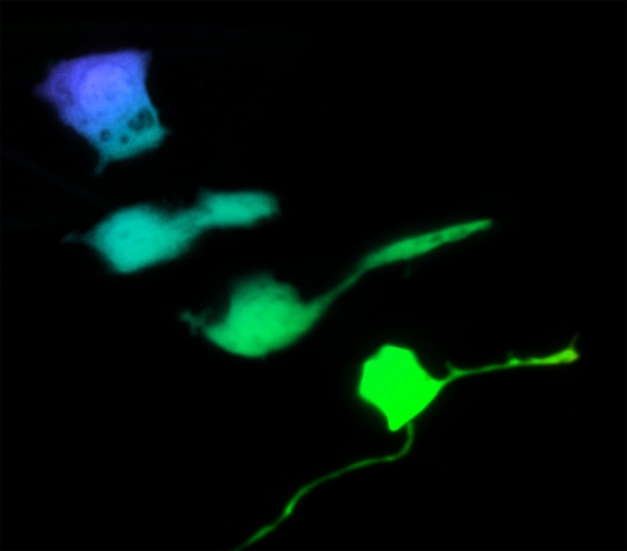
Development of the PQR oxygen sensory neuron in *C. elegans* proceeds with emergence of the dendrite from the posterior (right) side of the cell, as it senses an attractive source of ligand (Wnt molecule). Image credit: Leonie Kirszenblat, Divya Pattabiraman, and Luke Hammond.

[Fig pbio-1001154-g001]How does a dendrite grow? The answer to that question is central to understanding development of neurons, and thus to understanding entire nervous systems. But while the molecular signals underlying axon development have been relatively well described, comparatively little is known about the mechanisms of dendrite development, especially at its earliest stages. In this issue of *PLoS Biology*, Leonie Kirszenblat, Divya Pattabiraman, and Massimo Hilliard reveal a crucial role for a ligand-receptor pair that together coax the neuronal cell body to extend a dendrite toward its target.

The authors studied the so-called PQR neuron of the roundworm, *C. elegans*. PQR is an oxygen-sensing neuron located in the rear of the animal. One feature that makes it a good candidate for the study of neuronal development is its structural simplicity—it forms a single axon and a single dendrite, without the extensive branching that gives dendrites their name. Even more important for the current study, the neuron arises from its precursors post-embryonically, in the very early larval stage just after hatching, allowing the investigators to more easily observe influences on its development.

The members of the Wnt family of proteins are well known signaling molecules, and are important determinants of development in multicellular animals, and so the authors sought Wnt mutants that altered dendrite development. They identified one, called LIN-44, which, when mutated, led to shortened, absent, or misrouted dendrites, while having no effect on axons. LIN-44 had been previously shown to play roles in regulating neuronal polarity, axon guidance, and synapse formation. LIN-44 is produced not in the PQR neuron itself, but in cells in the tail near the final location of the PQR dendrite. This suggested that the dendrite may use LIN-44 as a target for its growth, thus serving an “instructive” role in dendrite growth. But an alternative possibility was that LIN-44 plays only a “permissive” role, merely triggering the dendrite to grow, while some other factor tells it where to go. To distinguish between these, the authors expressed LIN-44 in more anterior regions. When they did, the dendrite grew toward the head of the worm, indicating LIN-44 provided instruction, not just permission, for the growing dendrite.

That instructional signal is laid down early, even before the PQR cell itself appears. Even if the LIN-44 secreting cells were ablated shortly before PQR was born (while presumably leaving secreted LIN-44 in the surrounding tissue), the neuron still developed normally. But if expression of LIN-44 was delayed until after the dendrite began to grow, growth was abnormal, confirming the need for an early signal to the neuronal cell body.

LIN-17 is a known receptor for LIN-44, and mutation of LIN-17 created dendrite developmental defects similar to those of its ligand, indicating the two act in a single pathway. The authors found that LIN-17 was expressed weakly but ubiquitously on the neuronal membrane before dendrite outgrowth, which they suggest may help the neuron detect the directional signal from LIN-44. Another Wnt ligand, EGL-20, also participates in the dendrite development process alongside LIN-44, and genetic evidence indicates it may interact with LIN-17 as well.

Understanding these fundamental mechanisms of neuronal development may have practical, as well as theoretical, implications. Being able to control dendritic growth may be important for growing neurons from stem cells, which could find use in treatment of neurologic diseases such as spinal cord injury.

**Kirszenblat L, Pattabiraman D, Hilliard MA (2011) LIN-44/Wnt Directs Dendrite Outgrowth through LIN-17/Frizzled in** ***C. elegans*** **Neurons. doi:10.1371/journal.pbio.1001157**

